# The Relative Handgrip Strength and Risk of Cardiometabolic Disorders: A Prospective Study

**DOI:** 10.3389/fphys.2020.00719

**Published:** 2020-06-23

**Authors:** Guang Hao, Haiyan Chen, Yuting Ying, Min Wu, Guang Yang, Chunxia Jing

**Affiliations:** ^1^Department of Epidemiology, School of Medicine, Jinan University, Guangzhou, China; ^2^Department of Endemic Disease, Guangzhou Center for Disease Control and Prevention, Guangzhou, China; ^3^Department of Pathogen Biology, School of Medicine, Jinan University, Guangzhou, China; ^4^Guangdong Key Laboratory of Environmental Exposure and Health, Jinan University, Guangzhou, China

**Keywords:** handgrip strength, hypertension, diabetes, dyslipidemia, prospective study

## Abstract

**Background:**

This study aims to investigate the association between handgrip strength (HGS) and cardiometabolic disorders (CMD), including hypertension, diabetes, and dyslipidemia, in a prospective study.

**Methods:**

The association between HGS and CMD was examined using the data from 5,271 Chinese adult participants ≥45 years old enrolled in the CHARLS (Chinese Health and Retirement Prospective Cohort Study) during 2011–2015. Relative HGS, calculated as maximal absolute HGS from both hands divided by body mass index, was used in the primary analysis and divided into three groups according to the tertiles (T1, T2, and T3).

**Results:**

The participants with higher relative HGS had a lower risk of hypertension, diabetes, and dyslipidemia than those with lower HGS, although did not reach statistical significance for diabetes and hypertension in males. Participants with higher HGS had significantly lower risk of hypertension [T3 vs. T1: OR = 0.69, 95% CI = 0.51–0.91, *P* = 0.010] and dyslipidemia (OR = 0.65, 95% CI = 0.51–0.83, *P* < 0.001) in males. For females, participants with higher HGS had significantly lower risks of dyslipidemia (OR = 0.67, 95% CI = 0.54–0.83, *P* < 0.001).

**Conclusion:**

A consistent association was observed between higher relative HGS and lower risk of CMD. Further research is required to evaluate whether relative HGS can be a convincing predictor for the occurrence of CMD and as a target for intervention in the high-risk population.

## Introduction

Cardiometabolic disorders (CMD), including hypertension, diabetes, dyslipidemia, and cardiovascular disease, have been a leading cause of death in China and around the world (Yang Z.J. et al., 2012; GBD 2016 Causes of Death Collaborators, 2017; Lu et al., 2018). The trend of the burden from CMD is expected to continue increasing as economic progress, urbanization, and aging in China (Yang et al., 2013). Thus, early screening and health intervention efforts are essential to reduce the growing burden associated with the CMD.

Muscular fitness, as indicated by handgrip strength (HGS), has become an important cardiometabolic marker (Ramirez-Velez et al., 2016). HGS provides a simple and reliable surrogate of muscular strength and is considered as the most suitable indicator of muscular function in clinical settings (Buigues et al., 2015). Skeletal muscle is the largest insulin-sensitive tissue in the body and is the major site for insulin-stimulated glucose utilization (Stump et al., 2006); thus, loss of muscle mass and muscle quality may play a critical role in the development of CMD (Atlantis et al., 2009; Butcher et al., 2018; Kim and Park, 2018; Li et al., 2018). Although the association between HGS and CMD was found in several studies (Kelley and Kelley, 2010; Peterson et al., 2016, 2017; Karvonen-Gutierrez et al., 2018; Li et al., 2018; Zhang et al., 2018), most studies are cross-sectional design (Dong et al., 2016; Peterson et al., 2016, 2017; Ji et al., 2018; Li et al., 2018; Zhang et al., 2018; Wu et al., 2019) and controversial results exist (Dong et al., 2016; Pagonas et al., 2017; Giglio et al., 2018; Ji et al., 2018). For example, Peterson et al. (2017) analyzed the data from the U.S. National Health and Nutrition Examination Survey 2011–2012 and 2013–2014 combined surveys, and the 2011 wave of the China Health and Retirement Longitudinal Study, and found that normalized HGS was robustly associated with both CMD and physical disabilities in the United States and Chinese aging adults. Another cross-sectional data using the 2013 National Physical and Health in Shanxi Province involving 5,520 participants also found that HGS was associated with metabolic profiles and a higher risk of metabolic diseases (Li et al., 2018). A meta-analysis including 81 men and women (42 exercise and 39 controls) from three studies reported that isometric handgrip exercise is efficacious for reducing resting systolic blood pressure (BP) and diastolic BP in adults (Kelley and Kelley, 2010). However, Giglio et al. (2018) did not find low HGS normalized or not to body weight, was associated with hyperglycemia and diabetes in a population-based study. A study even reported a positive association between HGS with BP (Dong et al., 2016).

Therefore, this study aims to examine the longitudinal associations between HGS and CMD in a large cohort of Chinese population from the China Health and Retirement Longitudinal Study (CHARLS).

## Materials and Methods

### Study Ethics and Participants

The data were from CHARLS, a nationally representative longitudinal survey that examines the social, financial, and health circumstances of citizens in China. The details of the CHARLS study have been reported elsewhere (Zhao et al., 2014). Households were randomly chosen using a probability proportionate to size sampling strategy from 28 provinces in China at baseline in 2011. The participants aged over 45 years old and their spouses (if any, and of any age) were interviewed every 2 years. In this study, three times survey data (Wave2011, Wave2013, and Wave2015) during 2011–2015 were used. There were 10,071 respondents interviewed in 2011, and additional 4,215 new respondents were recruited in 2013. Eligible participants were those who entered the cohort in 2011 or 2013 and that at baseline were ≥45 (range: 45–93) years old, free of hypertension, diabetes, and dyslipidemia. A total of 2,883 participants were excluded due to missing data on HGS. We further excluded those that did not have data on hypertension, self-reported diabetes, and dyslipidemia (*N* = 1,478); had missing covariates on age, sex, education, marital status, smoking status, drinking status, body mass index (BMI), and physical activity at baseline (*N* = 3,829); and did not have at least one wave during their follow-up (*N* = 825). The final sample size was 5,271. The CHARLS study was approved by the Ethical Review Committee at Peking University (IRB00001052–11015).

### Measurements and Definitions

The data were collected by interviewers trained at Peking University. They interviewed respondents in their homes using CAPI (computer-assisted personal interview) technology. BMI was calculated as weight in kilograms divided by the square of height in meters. Current smokers were defined as smoking at least 1 cigarette per day and currently smoking, and former smokers were defined as those participants who reported that they had smoked at least 1 cigarette per day and were not currently smoking. Participants drinking any alcoholic beverages more than once a month in the past year were categorized as current drinkers, and former drinkers were defined as those participants drinking any alcoholic beverages more than once a month and were not currently drinking. Participants with vigorous or moderate physical activity were defined as undertaking vigorous or moderate activities at least 10 min per day and at least 3 days per week. Vigorous activities make you breathe much harder than normal and may include heavy lifting, digging, plowing, aerobics, fast bicycling, and cycling with a heavy load. Moderate physical activities make you breathe somewhat harder than normal and may include carrying light loads, bicycling at a regular pace, or mopping the floor. Marital status was dichotomized into currently married or not married (separated, divorced, widowed, never married or cohabitated). High school education or above means a 12-grade level or above, including high school, vocational school, college, or university.

The HGS (kg) was obtained by a dynamometer, measured with a standing position for a few seconds with the elbows at right angles. In the primary analysis, relative HGS, calculated as maximal absolute HGS from both hands divided by BMI (Lawman et al., 2016), was used for analyses and divided into three groups according to the tertiles (T1, T2, and T3) by sex. This index is a proxy for muscular strength independent of the influence of body mass. Meanwhile, the results of mean HGS and absolute HGS were also reported in the Supplementary Appendix. In addition, to facilitate comparison with previous studies, HGS was also divided into four groups according to the quartiles. According to the Chinese guidelines on prevention and treatment of dyslipidemia in adults (Joint committee for guideline revision, 2018), dyslipidemia was defined as total cholesterol (TC) ≥ 6.22 mmol/L (240 mg/dL), triglyceride (TG) ≥ 2.26 mmol/L (200 mg/dL), high-density lipoprotein cholesterol (HDLC) < 1.04 mmol/L (40 mg/dL), low-density lipoprotein cholesterol (LDLC) ≥ 4.14 mmol/L (160 mg/dL), or self-reported dyslipidemia. Hypertension was defined as individuals with systolic BP ≥ 140 mmHg and/or diastolic BP ≥ 90 mmHg or using antihypertensive drugs. Diabetes mellitus was defined as fasting glucose ≥7.0 mmol/L (≥126 mg/dL), glycohemoglobin (HbA1c) ≥ 6.5%, or using antidiabetic drugs.

### Statistical Analyses

Means or medians were calculated to present continuous variables and were compared by one-way analysis of variance (ANOVA) or appropriate nonparametric tests. Categorical variables were represented as a percentage and were tested by chi-square tests. The random-effect logistic regression (community–household–individual) was performed to examine the sex-stratified associations between HGS and CMD. Univariate analyses were performed in Model 1. Model 2 was adjusted for age. Model 3 was further adjusted for marital status, education levels, smoking, drinking, and physical activity. The odds ratios (ORs) and 95% confidence intervals (CIs) were calculated. Receiver operating characteristics (ROC) curves were performed to calculate the area under the curve (AUC), sensitivity, specificity, and cut-off points of HGS. In sensitivity analyses, we also investigated the associations between HGS and CMD using maximal absolute HGS and mean HGS of both hands (BMI was adjusted for in the models). In addition, 1,074 participants with self-reported stroke, chronic lung diseases, depressive symptoms (the 10-item Center for Epidemiological Studies–Depression Scale scored 12 points or more), or coronary heart diseases were excluded in the analyses. All analyses were performed using Stata software version 15.0 (STATA Corp., College Station, TX, United States). A two-sided *P* < 0.05 was considered statistically significant.

## Results

A total of 5,271 participants were eligible and included in the analysis. The baseline characteristics of the participants were presented according to the relative HGS tertiles (Table 1). Overall, the mean age was 57.6 years, and 46.7% were males. The average HGS was 32.3 kg. The mean follow-up was 3.7 years. The participants with higher HGS tend to be younger age, married, and lower BMI (*P* < 0.001).

**TABLE 1:** Characteristics of all participants by relative handgrip strength groups*.

Characteristics	Males (*N* = 2,481)				Females (*N* = 2,790)			
	T1	T2	T3	*P* value	T1	T2	T3	*P* value
Follow-up period (year)	3.8 ± 0.6	3.7 ± 0.7	3.7 ± 0.7	0.600	3.7 ± 0.7	3.7 ± 0.7	3.7 ± 0.7	0.500
Age, years (%)	62.3 ± 9.3	58.5 ± 8.4	55.2 ± 7.2	<0.001	59.2 ± 8.8	55.8 ± 7.7	54.6 ± 7.6	<0.001
45–55	197 (23.8)	303 (36.6)	432 (52.2)	<0.001	318 (34.2)	462 (49.7)	518 (55.7)	<0.001
55–65	321 (38.8)	345 (41.7)	309 (37.4)	–	379 (40.8)	364 (39.1)	313 (33.7)	–
≥65	309 (37.4)	179 (21.6)	86 (10.4)	–	233 (25.1)	104 (11.2)	99 (10.6)	–
High school education or above (%)	159 (19.2)	186 (22.5)	230 (27.8)	<0.001	175 (18.8)	187 (20.1)	177 (19.0)	0.752
Married (%)	738 (89.2)	766 (92.6)	776 (93.8)	0.002	802 (86.2)	838 (90.1)	846 (91.0)	0.002
**Smokers (%)**								
Never	267 (32.3)	210 (25.4)	183 (22.1)	<0.001	858 (92.3)	865 (93)	857 (92.2)	0.751
Former	125 (15.1)	123 (14.9)	115 (13.9)	–	14 (1.5)	18 (1.9)	16 (1.7)	–
Current	435 (52.6)	494 (59.7)	529 (64.0)	–	58 (6.2)	47 (5.1)	57 (6.1)	–
**Drinkers (%)**								
Never	396 (47.9)	378 (45.7)	365 (44.1)	<0.001	823 (88.5)	837 (90)	849 (91.3)	0.138
Former	102 (12.3)	69 (8.3)	59 (7.1)	–	30 (3.2)	19 (2.0)	15 (1.6)	–
Current	329 (39.8)	380 (45.9)	403 (48.7)	–	77 (8.3)	74 (8.0)	66 (7.1)	–
Vigorous/moderate physical activity (%)	203 (24.5)	230 (27.8)	228 (27.6)	0.247	214 (23.0)	298 (32.0)	291 (31.3)	<0.001
Handgrip strength (kg)	30.3 ± 6.6	38.6 ± 5.1	45.8 ± 7.8	<0.001	19.9 ± 4.8	26.5 ± 3.7	32.5 ± 5.7	<0.001
Relative handgrip strength	1.3 ± 02	1.7 ± 0.1	2.2 ± 0.3	<0.001	0.8 ± 0.2	1.1 ± 0.1	1.5 ± 0.2	<0.001
Body mass index (kg/m^2^)	23.6 ± 3.9	22.4 ± 2.8	21.3 ± 2.5	<0.001	24.8 ± 4.2	23.7 ± 3.2	22.1 ± 3.0	<0.001

The means and standard deviations (SDs) were reported for continuous data, and the frequencies and percentages were reported for categorical data. *Relative handgrip strength was calculated as maximal absolute handgrip strength from both hands divided by body mass index, and was divided into three groups according to the tertiles (T1, T2, and T3 group).

The incidence of CMD was higher in the population with lower HGS, although did not reach statistical significance for diabetes and hypertension in males (Tables 2, 3). The adjusted OR of T2 group for hypertension was 1.07 (95% CI = 0.84–1.39, *P* = 0.562), and T3 group was 0.69 (95% CI = 0.51–0.91, *P* = 0.010) in males (*P* for trend = 0.015). For females, the adjusted OR of T2 group was 0.84 (95% CI = 0.25–2.85, *P* = 0.780), and T3 group was 0.59 (95% CI = 0.15–2.29, *P* = 0.448) (*P* for trend = 0.450). Furthermore, the participants with higher HGS had a lower level of BP (Supplementary Table 1).

**TABLE 2:** The associations between relative handgrip strength and cardiometabolic disorders in males.

Grip strength	Hypertension			Diabetes			Dyslipidemia		
	*N* (%)	OR (95% CI)	*P* value	*N* (%)	OR (95% CI)	*P* value	*N* (%)	OR (95% CI)	*P* value
**Model 1**									
Tertile 1	188 (22.7)	Reference	–	98 (11.9)	Reference	–	254 (30.7)	Reference	–
Tertile 2	184 (22.3)	0.97(0.76–1.23)	0.782	65 (7.9)	0.62(0.10–3.80)	0.605	247 (30.0)	0.95(0.77–1.18)	0.662
Tertile 3	120 (14.5)	0.56(0.43–0.74)	<0.001	53 (6.4)	0.49(0.07–3.41)	0.474	211 (25.5)	0.76(0.61–0.95)	0.017
*P* for trend	–	<0.001	–	–	0.460	–	–	0.018	–
**Model 2**									
Tertile 1	188 (22.7)	Reference	–	98 (11.9)	Reference	–	254 (30.7)	Reference	–
Tertile 2	184 (22.3)	1.07(0.83–1.37)	0.599	65 (7.9)	0.63(0.10–4.01)	0.626	247 (30.0)	0.87(0.70–1.09)	0.235
Tertile 3	120 (14.5)	0.68(0.51–0.90)	0.008	53 (6.4)	0.51(0.07–3.93)	0.518	211 (25.5)	0.65(0.51–0.82)	<0.001
*P* for trend	–	0.011	–	–	0.506	–	–	<0.001	–
**Model 3**									
Tertile 1	188 (22.7)	Reference	–	98 (11.9)	Reference	–	254 (30.7)	Reference	–
Tertile 2	184 (22.3)	1.07(0.84–1.39)	0.562	65 (7.9)	0.63(0.10–4.05)	0.630	247 (30.0)	0.88(0.70–1.10)	0.248
Tertile 3	120 (14.5)	0.69(0.51–0.91)	0.010	53 (6.4)	0.51(0.07–3.99)	0.521	211 (25.5)	0.65(0.51–0.83)	<0.001
*P* for trend	–	0.015	–	–	0.509	–	–	<0.001	–

*Model 1: Unadjusted; Model 2: Adjusted for age; Model 3: Adjusted for age, marital status, education levels, smoking, drinking, and vigorous or moderate physical activity.*

**TABLE 3:** The associations between relative handgrip strength and cardiometabolic disorders in females.

Grip strength	Hypertension			Diabetes			Dyslipidemia		
	*N* (%)	OR (95% CI)	*P* value	*N* (%)	OR (95% CI)	*P* value	*N* (%)	OR (95% CI)	*P* value
**Model 1**									
Tertile 1	192 (20.7)	Reference	–	216 (12.3)	Reference	–	552 (31.4)	Reference	–
Tertile 2	153 (16.5)	0.74(0.22–2.45)	0.618	152 (8.7)	0.54(0.11–2.57)	0.442	484 (27.6)	0.87(0.70–1.09)	0.235
Tertile 3	110 (11.8)	0.50(0.13–1.86)	0.299	122 (6.9)	0.40(0.07–2.23)	0.294	465 (26.5)	0.65(0.51–0.82)	<0.001
*P* for trend	–	0.295	–	–	0.270	–	–	0.001	–
**Model 2**									
Tertile 1	192 (20.7)	Reference	–	216 (12.3)	Reference	–	552 (31.4)	Reference	–
Tertile 2	153 (16.5)	0.84(0.25–2.84)	0.779	152 (8.7)	0.60(0.12–2.88)	0.519	484 (27.6)	0.90(0.73–1.10)	0.291
Tertile 3	110 (11.8)	0.60(0.15–2.31)	0.456	122 (6.9)	0.45(0.08–2.60)	0.372	465 (26.5)	0.66(0.53–0.83)	<0.001
*P* for trend	–	0.458	–	–	0.351	–	–	<0.001	–
**Model 3**									
Tertile 1	192 (20.7)	Reference	–	122 (13.1)	Reference	–	290 (31.2)	Reference	–
Tertile 2	153 (16.5)	0.84(0.25–2.85)	0.780	92 (9.9)	0.60(0.12–2.94)	0.533	274 (29.5)	0.91(0.74–1.12)	0.373
Tertile 3	110 (11.8)	0.59(0.15–2.29)	0.448	60 (6.5)	0.44(0.08–2.61)	0.369	225 (24.2)	0.67(0.54–0.83)	<0.001
*P* for trend	–	0.450	–	–	0.350	–	–	<0.001	–

*Model 1: Unadjusted; Model 2: Adjusted for age; Model 3: Adjusted for age, marital status, education levels, smoking, drinking, and vigorous or moderate physical activity.*

The adjusted OR of T2 group for diabetes was 0.63 (95% CI = 0.10–4.05, *P* = 0.630), and T3 group was 0.51 (95% CI = 0.07–3.99, *P* = 0.521) in males (*P* for trend = 0.509). The adjusted OR of T2 group was 0.60 (95% CI = 0.12–2.94, *P* = 0.533), and T3 was 0.44 (95% CI = 0.08–2.61, *P* = 0.369) in females (*P* for trend = 0.350). In addition, the stronger HGS was significantly associated with the lower level of fasting blood glucose and HbA1c (*P* for trend <0.001) (Supplementary Table 2).

The adjusted OR of T2 group for dyslipidemia was 0.88 (95% CI = 0.70–1.10, *P* = 0.248), and T3 group was 0.65 (95% CI = 0.51–0.83, *P* < 0.001) in males (*P* for trend <0.001). For females, the adjusted OR of T2 group was 0.91 (95% CI = 0.74–1.12, *P* = 0.373), and T3 group was 0.67 (95% CI = 0.54–0.83, *P* < 0.001) (*P* for trend <0.001). Additionally, the stronger HGS was significantly associated with the lower level of HDLC and TG (*P* for trend <0.001) (Supplementary Table 3).

Sensitivity, specificity, AUC, and cut-off points of relative HGS were reported in Supplementary Table 4. Sensitivity analyses using mean relative HGS found similar results. However, we did not find an statistical association between absolute HGS and CMD except for dyslipidemia (Supplementary Table 5). Furthermore, HGS was divided into four groups according to the quartiles, and the results were shown in Supplementary Table 6. In addition, after excluding 1,074 participants with self-reported stroke, chronic lung diseases, depressive symptoms, or coronary heart diseases, the results remain broadly the same (Supplementary Table 7).

## Discussion

The present data have demonstrated that low relative HGS was longitudinally associated with an increased risk of hypertension, diabetes, and dyslipidemia based on a large population-based sample from China. This finding suggests that relative HGS may be an early target for intervention or a marker to identify people at risk of CMD.

A growing body of research has demonstrated an association between decreased HGS, assessed using varied indices such as the absolute HGS, HGS normalized to body weight and HGS normalized to BMI, and a higher risk of CMD, but most studies were conducted using a cross-sectional design. Although most evidence indicated a negative association between HGS and hypertension or BP (Sayer et al., 2007; Inder et al., 2016; Kawamoto et al., 2016), the results are still mixed (Ash et al., 2017). A cross-sectional study within the Hertfordshire Cohort Study demonstrated that impaired HGS was associated with the metabolic syndrome, and the individual components, including fasting TG, BP, waist circumference, 2-h glucose, and homeostasis model assessment-estimated insulin resistance (Sayer et al., 2007). A meta-analysis including 11 randomized trials and totaling 302 participants suggested that isometric resistance training may elicit BP reductions greater than those seen with dynamic aerobic and resistance exercise (Inder et al., 2016), although the results continue to be controversial (Ash et al., 2017). Our study supported previous studies that decreased relative HGS was significantly associated with a higher risk of hypertension or BP level.

Few studies to date have examined the longitudinal association of HGS with diabetes (Wander et al., 2011; Leong et al., 2015), and results are mixed (Derave et al., 2003; Cetinus et al., 2005; Sayer et al., 2005; Park et al., 2006; Wander et al., 2011; Leong et al., 2015; van der Kooi et al., 2015; Marques-Vidal et al., 2017). Wander et al. reported that higher absolute HGS was associated with a lower risk of developing type 2 diabetes in lean Japanese American individuals (Wander et al., 2011). The Healthy Life in an Urban Setting study also reported an inverse association between absolute HGS and type 2 diabetes in a cross-sectional study, and this association did not differ substantially among all ethnic groups, among both sexes, and among lean and overweight individuals (van der Kooi et al., 2015). Even within the normal range of blood glucose, a study also reported that the higher glucose level is associated with lower HGS (Sayer et al., 2005). Case-control studies also reported that diabetes was associated with lower skeletal muscle strength and quality (Cetinus et al., 2005; Park et al., 2006). Furthermore, a clinical trial showed that resistance exercise training could improve glucose tolerance and enhance insulin action in skeletal muscle (Derave et al., 2003). After a median follow-up of 4.0 years, the Prospective Urban–Rural Epidemiology (PURE) study found that absolute HGS was inversely associated with all-cause death, cardiovascular death, and cardiovascular disease, but found no significant association between HGS and incident diabetes (Leong et al., 2015). In addition, there seems to be a bidirectional link between HGS and incident diabetes (Mainous et al., 2015). Our findings are in line with most previous findings that HGS was negatively associated with risk of diabetes, although did not reach statistical significance.

The association between HGS and lipid profile seems more complex. On multivariate analysis, we found that lower relative HGS was significantly associated with higher dyslipidemia and components (HDLC and TG). For TC and LDLC, we only found a significant association with the moderate HGS group, suggesting an inverse U-shaped association between grip strength and TC and LDLC (although we did not find a significant U-shaped association, and data not shown), which was in line with the CoLaus study (the city of Lausanne, Switzerland) (Gubelmann et al., 2017). These findings seem to be in agreement with previous studies (Kawamoto et al., 2016; Lee et al., 2016) but not with another (Giglio et al., 2018). A study of 927 older people in Taiwan found that relative HGS was significantly associated with a favorable lipid profile, including TG, TC/HDLC ratio (Lee et al., 2016). Kawamoto et al. (2016) showed that weight-adjusted HGS was significantly associated with a favorable lipid profile of TC and HDLC, but not LDLC. However, a cross-sectional study reported that low HGS normalized or not to body weight was not associated with hyperglycemia (Giglio et al., 2018).

It implied that relative HGS, but not absolute HGS, was a more appropriate instrument for the prediction of CMD. Some previous studies failed to demonstrate the association between cardiometabolic risk and HGS, which may have resulted from using absolute HGS instead of relative HGS (Aoyama et al., 2011; Yang E.J. et al., 2012; Liu et al., 2014; Gubelmann et al., 2017; Byeon et al., 2019). Our results were in line with one previous study in which relative HGS and favorable cardiometabolic risk factors including BP, TG, TC/HDL-C ratio, HbA1c, uric acid, but dominant HGS was not significantly associated with all cardiovascular biomarkers and Framingham Risk Score in both sexes (Lee et al., 2016). Another study of older Japanese in the rural area using weight-adjusted HGS also showed that the association between lower cardiometabolic risk and higher HGS, and relative HGS, instead of dominant/absolute HGS, was significantly associated with metabolic syndrome (Kawamoto et al., 2016). A population-based survey performed in China also demonstrated that relative HGS was associated with a favorable metabolic profile and a lower risk of metabolic disease; however, higher absolute HGS was associated with unfavorable metabolic profiles and a higher risk of metabolic diseases (Li et al., 2018).

HGS measurement may appeal as a quick and inexpensive way to identify the high-risk population, but the potential mechanism linking low HGS to the CMD is not fully understood. A study indicated that physical activity could partly explain the association between HGS and glucose levels. However, the association remained significant after adjustment for physical activity, suggesting that the underlying mechanisms are not fully explained (Sayer et al., 2005). Unlike aerobic training, strength training has been observed to have a protective effect on HbA1c and lipid profiles (Yuing Farias et al., 2015). In overweight and obese subjects, strength training improved insulin sensitivity and lipid profile without any change in body mass (Hernan Jimenez and Ramirez-Velez, 2011). Steene-Johannessen et al. (2009) reported that higher cardiorespiratory fitness and muscle fitness were associated with lower levels of chronic inflammation markers that were considered playing an essential role in CMD (Donath et al., 2019). Muscle fitness has also been shown to have a positive influence on cardiometabolic risk, independent of cardiorespiratory fitness (Artero et al., 2011, 2012). This independent influence maybe because of distinct cellular signaling pathways in response to strength training and endurance training (Hawley, 2009). Also, both sarcopenia and insulin-resistant states are associated with the accumulation of myofibre lipids, indicating that they may share common cellular and molecular changes (Sayer et al., 2007).

One major strength was that we reported the longitudinal association between HGS and CMD in a large Chinese population. However, there were several limitations in our study. First, we cannot fully exclude the possibility that the observed association is due to residual confoundings, such as diet and genetic factors, which reported to be factors that may influence the progress in CMD. Second, this study had a relatively short follow-up period. Third, The CHARLS study did not collect the data on lipid-lowering medications. However, we considered self-reported dyslipidemia in the definition of dyslipidemia. Finally, the sex differences should be further studied.

## Conclusion

Our findings reported a negative association between relative HGS and CMD, suggesting that HGS may be an early predictor of CMD. Further mechanistic studies and clinical trials are needed to discover whether HGS can be a convincing predictor for the occurrence of CMD and as a target for intervention at high-risk population.

## Data Availability Statement

The datasets generated for this study are available at: http://charls.pku.edu.cn/index/en.html.

## Ethics Statement

The studies involving human participants were reviewed and approved by Ethical Review Committee at Peking University. The patients/participants provided their written informed consent to participate in this study.

## Author Contributions

GH and CJ designed the study, had full access to all the data in the study, and took responsibility for the integrity of the data and the accuracy of the data analyses. GH planned and did the analyses. GH and HC drafted the initial manuscript. All authors contributed to the revising of the manuscript.

## Conflict of Interest

The authors declare that the research was conducted in the absence of any commercial or financial relationships that could be construed as a potential conflict of interest.

## References

[B1] Aoyama T. Asaka M. Ishijima T. Kawano H. Cao Z. B. Sakamoto S. (2011). Association between muscular strength and metabolic risk in Japanese women, but not in men. *J. Physiol. Anthropol.* 30 133–139. 10.2114/jpa2.30.133 21804295

[B2] Artero E. G. Lee D. C. Lavie C. J. Espana-Romero V. Sui X. Church T. S. (2012). Effects of muscular strength on cardiovascular risk factors and prognosis. *J. Cardiopul. Rehabil. Prevent.* 32 351–358. 10.1097/hcr.0b013e3182642688 22885613 PMC3496010

[B3] Artero E. G. Ruiz J. R. Ortega F. B. Espana-Romero V. Vicente-Rodriguez G. Molnar D. (2011). Muscular and cardiorespiratory fitness are independently associated with metabolic risk in adolescents: the HELENA study. *Pediatr. Diabetes* 12 704–712. 10.1111/j.1399-5448.2011.00769.x 21470352

[B4] Ash G. I. Taylor B. A. Thompson P. D. MacDonald H. V. Lamberti L. Chen M. H. (2017). The antihypertensive effects of aerobic versus isometric handgrip resistance exercise. *J. Hypertens.* 35 291–299. 10.1097/hjh.0000000000001176 27861249 PMC5713890

[B5] Atlantis E. Martin S. A. Haren M. T. Taylor A. W. Wittert G. A. (2009). Inverse associations between muscle mass, strength, and the metabolic syndrome. *Metab. Clin. Exp.* 58 1013–1022. 10.1016/j.metabol.2009.02.027 19394973

[B6] Buigues C. Padilla-Sanchez C. Garrido J. F. Navarro-Martinez R. Ruiz-Ros V. Cauli O. (2015). The relationship between depression and frailty syndrome: a systematic review. *Aging Ment. Health* 19 762–772. 10.1080/13607863.2014.967174 25319638

[B7] Butcher J. T. Mintz J. D. Larion S. Qiu S. Ruan L. Fulton D. J. (2018). Increased muscle mass protects against hypertension and renal injury in obesity. *J. Am. Heart Assoc.* 7:e009358. 10.1161/JAHA.118.009358 PMC6201396 30369309

[B8] Byeon J. Y. Lee M. K. Yu M. S. Kang M. J. Lee D. H. Kim K. C. (2019). Lower relative handgrip strength is significantly associated with a higher prevalence of the metabolic syndrome in adults. *Metab. Syndr. Relat. Disord.* 17 280–288. 10.1089/met.2018.0111 30945974

[B9] Cetinus E. Buyukbese M. A. Uzel M. Ekerbicer H. Karaoguz A. (2005). Hand grip strength in patients with type 2 diabetes mellitus. *Diabetes. Res. Clin. Pract.* 70 278–286. 10.1016/j.diabres.2005.03.028 15878215

[B10] Derave W. Eijnde B. O. Verbessem P. Ramaekers M. Van Leemputte M. Richter E. A. (2003). Combined creatine and protein supplementation in conjunction with resistance training promotes muscle GLUT-4 content and glucose tolerance in humans. *J. Appl. Physiol.* 94 1910–1916. 10.1152/japplphysiol.00977.2002 12524381

[B11] Donath M. Y. Meier D. T. Boni-Schnetzler M. (2019). Inflammation in the pathophysiology and therapy of cardiometabolic disease. *Endocr. Rev.* 40 1080–1091. 10.1210/er.2019-00002 31127805 PMC6624792

[B12] Dong B. Wang Z. Arnold L. Song Y. Wang H. J. Ma J. (2016). The association between blood pressure and grip strength in adolescents: does body mass index matter? *Hypertens.Res.* 39 919–925. 10.1038/hr.2016.84 27383511

[B13] GBD 2016 Causes of Death Collaborators (2017). Global, regional, and national age-sex specific mortality for 264 causes of death, 1980-2016: a systematic analysis for the global burden of disease study 2016. *Lancet* 390 1151–1210. 28919116 10.1016/S0140-6736(17)32152-9 PMC5605883

[B14] Giglio B. M. Mota J. F. Wall B. T. Pimentel G. D. (2018). Low handgrip strength is not associated with type 2 diabetes mellitus and hyperglycemia: a population-based study. *Clin. Nutr. Res.* 7 112–116. 29713619 10.7762/cnr.2018.7.2.112 PMC5921328

[B15] Gubelmann C. Vollenweider P. Marques-Vidal P. (2017). Association of grip strength with cardiovascular risk markers. *Eur. J. Prevent. Cardiol.* 24 514–521. 10.1177/2047487316680695 27885059

[B16] Hawley J. A. (2009). Molecular responses to strength and endurance training: are they incompatible? *Appl. Physiol. Nutr. Metab.* 34 355–361. 10.1139/h09-023 19448698

[B17] Hernan Jimenez O. Ramirez-Velez R. (2011). Strength training improves insulin sensitivity and plasma lipid levels without altering body composition in overweight and obese subjects. *Endocrinol. Nutr.* 58 169–174. 10.1016/s2173-5093(11)70041-1 21459690

[B18] Inder J. D. Carlson D. J. Dieberg G. McFarlane J. R. Hess N. C. Smart N. A. (2016). Isometric exercise training for blood pressure management: a systematic review and meta-analysis to optimize benefit. *Hypertens. Res.* 39 88–94. 10.1038/hr.2015.111 26467494

[B19] Ji C. Zheng L. Zhang R. Wu Q. Zhao Y. (2018). Handgrip strength is positively related to blood pressure and hypertension risk: results from the National Health and nutrition examination survey. *Lipids Health Dis.* 17:86. 10.1186/s12944-018-0734-4 PMC5904981 29665844

[B20] Joint committee for guideline revision (2018). 2016. Chinese guidelines for the management of dyslipidemia in adults. *J. Geriatr.Cardiol.* 15 1–29. 29434622 10.11909/j.issn.1671-5411.2018.01.011 PMC5803534

[B21] Karvonen-Gutierrez C. A. Peng Q. Peterson M. Duchowny K. Nan B. Harlow S. (2018). Low grip strength predicts incident diabetes among mid-life women: the michigan study of women’s health across the nation. *Age. Ageing* 47 685–691. 10.1093/ageing/afy067 29726885 PMC6108393

[B22] Kawamoto R. Ninomiya D. Kasai Y. Kusunoki T. Ohtsuka N. Kumagi T. (2016). Handgrip strength is associated with metabolic syndrome among middle-aged and elderly community-dwelling persons. *Clin. Exp. Hypertens.* 38 245–251. 10.3109/10641963.2015.1081232 26818203

[B23] Kelley G. A. Kelley K. S. (2010). Isometric handgrip exercise and resting blood pressure: a meta-analysis of randomized controlled trials. *J. Hypertens.* 28 411–418. 10.1097/hjh.0b013e3283357d16 20009767

[B24] Kim K. Park S. M. (2018). Association of muscle mass and fat mass with insulin resistance and the prevalence of metabolic syndrome in Korean adults: a cross-sectional study. *Sci. Rep.* 8:2703. 10.1038/s41598-018-21168-5 PMC5807388 29426839

[B25] Lawman H. G. Troiano R. P. Perna F. M. Wang C. Y. Fryar C. D. Ogden C. L. (2016). Associations of relative handgrip strength and cardiovascular disease biomarkers in U.S. Adults, 2011-2012. *Am. J. Prevent. Med.* 50 677–683. 10.1016/j.amepre.2015.10.022 26689977 PMC7337414

[B26] Lee W. J. Peng L. N. Chiou S. T. Chen L. K. (2016). Relative handgrip strength is a simple indicator of cardiometabolic risk among middle-aged and older people: a nationwide population-based study in taiwan. *PLoS One* 11:e0160876. 10.1371/journal.pone.0160876 27559733 PMC4999244

[B27] Leong D. P. Teo K. K. Rangarajan S. Lopez-Jaramillo P. Avezum A. Jr. Orlandini A. (2015). Prognostic value of grip strength: findings from the prospective urban rural epidemiology (PURE) study. *Lancet* 386 266–273. 10.1016/s0140-6736(14)62000-6 25982160

[B28] Li D. Guo G. Xia L. Yang X. Zhang B. Liu F. (2018). Relative handgrip strength is inversely associated with metabolic profile and metabolic disease in the general population in China. *Front. Physiol.* 9:59. 10.3389/fphys.2018.00059 29459831 PMC5807728

[B29] Liu L. K. Lee W. J. Chen L. Y. Hwang A. C. Lin M. H. Peng L. N. (2014). Sarcopenia, and its association with cardiometabolic and functional characteristics in Taiwan: results from I-Lan longitudinal aging study. *Geriatr. Gerontol Int.* 14(Suppl. 1), 36–45. 10.1111/ggi.12208 24450559

[B30] Lu Y. Wang P. Zhou T. Lu J. Spatz E. S. Nasir K. (2018). Comparison of prevalence, awareness, treatment, and control of cardiovascular risk factors in China and the United States. *J. Am. Heart Assoc.* 7:e007462. 10.1161/JAHA.117.007462 PMC5850247 29374046

[B31] Mainous A. G. Tanner R. J. Anton S. D. Jo A. (2015). Grip strength as a marker of hypertension and diabetes in healthy Weight Adults. *Am. J. Prev. Med.* 49 850–858. 10.1016/j.amepre.2015.05.025 26232901 PMC4656117

[B32] Marques-Vidal P. Vollenweider P. Waeber G. Jornayvaz F. R. (2017). Grip strength is not associated with incident type 2 diabetes mellitus in healthy adults: the CoLaus study. *Diabetes. Res. Clin. Pract.* 132 144–148. 10.1016/j.diabres.2017.08.004 28863331

[B33] Pagonas N. Vlatsas S. Bauer F. Seibert F. S. Zidek W. Babel N. (2017). Aerobic versus isometric handgrip exercise in hypertension: a randomized controlled trial. *J. Hypertens.* 35 2199–2206. 10.1097/hjh.0000000000001445 28622156

[B34] Park S. W. Goodpaster B. H. Strotmeyer E. S. de Rekeneire N. Harris T. B. Schwartz A. V. (2006). Decreased muscle strength and quality in older adults with type 2 diabetes: the health, aging, and body composition study. *Diabetes Metab. Res. Rev.* 55 1813–1818. 10.2337/db05-1183 16731847

[B35] Peterson M. D. Duchowny K. Meng Q. Wang Y. Chen X. Zhao Y. (2017). Low normalized grip strength is a biomarker for cardiometabolic disease and physical disabilities among U.S. and Chinese Adults. *J. Gerontol. Ser. A Biol. Sci. Med. Sci.* 72 1525–1531. 10.1093/gerona/glx031 28329157 PMC5861974

[B36] Peterson M. D. McGrath R. Zhang P. Markides K. S. Al Snih S. Wong R. (2016). Muscle weakness is associated with diabetes in older mexicans: the mexican health and aging study. *J. Am. Med. Dir. Assoc.* 17 933–938. 10.1016/j.jamda.2016.06.007 27450948 PMC5039066

[B37] Ramirez-Velez R. Correa-Bautista J. E. Lobelo F. Izquierdo M. Alonso-Martinez A. Rodriguez-Rodriguez F. (2016). High muscular fitness has a powerful protective cardiometabolic effect in adults: influence of weight status. *BMC Public Health* 16:1012. 10.1186/s12889-016-3678-5 27663845 PMC5035511

[B38] Sayer A. A. Dennison E. M. Syddall H. E. Gilbody H. J. Phillips D. I. Cooper C. (2005). Type 2 diabetes, muscle strength, and impaired physical function: the tip of the iceberg? *Diabetes Care* 28 2541–2542. 10.2337/diacare.28.10.2541 16186295

[B39] Sayer A. A. Syddall H. E. Dennison E. M. Martin H. J. Phillips D. I. Cooper C. (2007). Grip strength and the metabolic syndrome: findings from the Hertfordshire Cohort Study. *QJMMonth. J. Assoc. Phys.* 100 707–713. 10.1093/qjmed/hcm095 17951315 PMC2292249

[B40] Steene-Johannessen J. Anderssen S. A. Kolle E. Andersen L. B. (2009). Low muscle fitness is associated with metabolic risk in youth. *Med. Sci. Sports Exerc.* 41 1361–1367. 10.1249/mss.0b013e31819aaae5 19516166

[B41] Stump C. S. Henriksen E. J. Wei Y. Sowers J. R. (2006). The metabolic syndrome: role of skeletal muscle metabolism. *Ann. Med.* 38 389–402. 10.1080/07853890600888413 17008303

[B42] van der Kooi A. L. Snijder M. B. Peters R. J. van Valkengoed I. G. (2015). The Association of handgrip strength and type 2 diabetes mellitus in six ethnic groups: an analysis of the HELIUS study. *PLoS One* 10:e0137739. 10.1371/journal.pone.0137739 26368020 PMC4569584

[B43] Wander P. L. Boyko E. J. Leonetti D. L. McNeely M. J. Kahn S. E. Fujimoto W. Y. (2011). Greater hand-grip strength predicts a lower risk of developing type 2 diabetes over 10 years in leaner Japanese Americans. *Diabetes. Res. Clin. Pract.* 92 261–264. 10.1016/j.diabres.2011.01.007 21281974 PMC3910507

[B44] Wu H. Liu M. Chi V. T. Q. Wang J. Zhang Q. Liu L. (2019). Handgrip strength is inversely associated with metabolic syndrome and its separate components in middle aged and older adults: a large-scale population-based study. *Metabolism* 93 61–67. 10.1016/j.metabol.2019.01.011 30690038

[B45] Yang E. J. Lim S. Lim J. Y. Kim K. W. Jang H. C. Paik N. J. (2012). Association between muscle strength and metabolic syndrome in older Korean men and women: the Korean Longitudinal Study on Health and Aging. *Metab. Clin. Exp.* 61 317–324. 10.1016/j.metabol.2011.07.005 21871640

[B46] Yang Z. J. Liu J. Ge J. P. Chen L. Zhao Z. G. Yang W. Y. (2012). Prevalence of cardiovascular disease risk factor in the Chinese population: the 2007-2008 China National Diabetes and Metabolic Disorders Study. *Eur. Heart J.* 33 213–220. 10.1093/eurheartj/ehr205 21719451

[B47] Yang G. Wang Y. Zeng Y. Gao G. F. Liang X. Zhou M. (2013). Rapid health transition in China, 1990-2010: findings from the global burden of disease study 2010. *Lancet* 381 1987–2015. 10.1016/s0140-6736(13)61097-1 23746901 PMC7159289

[B48] Yuing Farias T. Santos-Lozano A. Solis Urra P. Cristi-Montero C. (2015). Effects of training and detraining on glycosylated haemoglobin, glycaemia and lipid profile in Type-Ii Diabetics. *Nutr. Hosp.* 32 1729–1734. 26545543 10.3305/nh.2015.32.4.9341

[B49] Zhang R. Li C. Liu T. Zheng L. Li S. (2018). Handgrip strength and blood pressure in children and adolescents: evidence from NHANES 2011 to 2014. *Am. J. Hypertens* 31 792–796. 10.1093/ajh/hpy032 29529209 PMC5998948

[B50] Zhao Y. Hu Y. Smith J. P. Strauss J. Yang G. (2014). Cohort profile: the China health and retirement longitudinal study (CHARLS). *Int. J. Epidemiol.* 43 61–68. 10.1093/ije/dys203 23243115 PMC3937970

