# Supplementary material for: The Relative Handgrip Strength and Risk of Cardiometabolic Disorders: A Prospective Study
Source: Front Physiol. 2020 Jun 23;11:719. doi: 10.3389/fphys.2020.00719 (PMC7344191; doi:10.3389/fphys.2020.00719)
Supplement: Supplementary file 1 [file Table_1.docx]

Supplementary Appendix

| Supplementary Table 1. The associations between relative handgrip strength and hypertension | | | | |
| --- | --- | --- | --- | --- |
| Grip strength | Systolic blood pressure | | Diastolic blood pressure | |
|  | β(95%CI) | *P* value | β(95%CI) | *P* value |
| **Males** |  |  |  |  |
| Tertile 1 | Reference |  | Reference |  |
| Tertile 2 | 0.60(-4.61,5.82) | 0.820 | -0.15(-1.16,0.87) | 0.780 |
| Tertile 3 | -2.38(-7.83,3.08) | 0.393 | -2.04(-3.13,-0.94) | <0.001 |
| *P* for trend | 0.392 |  | <0.001 |  |
| **Females** |  |  |  |  |
| Tertile 1 | Reference |  | Reference |  |
| Tertile 2 | -6.42(-10.66,-2.18) | 0.003 | -1.13(-2.06,-0.20) | 0.017 |
| Tertile 3 | -4.79(-9.16,-0.42) | 0.032 | -2.17(-3.15,-1.19) | <0.001 |
| *P* for trend | 0.037 |  | <0.001 |  |

Adjusted for age, marital status, education levels, smoking, drinking, and vigorous or moderate physical activity.

| Supplementary Table 2. The associations between relative handgrip strength and glucose metabolism | | | | |
| --- | --- | --- | --- | --- |
| Grip strength | Glucose | Glycohemoglobin | | |
|  | β(95%CI) | *P* value | β(95%CI) | *P* value |
| **Males** |  |  |  |  |
| Tertile 1 | Reference |  | Reference |  |
| Tertile 2 | -4.34(-6.98,-1.70) | 0.001 | -0.07(-0.14,0.00) | 0.057 |
| Tertile 3 | -5.04(-7.84,-2.25) | <0.001 | -0.10(-0.18,-0.03) | 0.005 |
| *P* for trend | <0.001 |  | 0.005 |  |
| **Females** |  |  |  |  |
| Tertile 1 | Reference |  | Reference |  |
| Tertile 2 | -3.88(-6.05,-1.70) | <0.001 | -0.08(-0.15,-0.02) | 0.010 |
| Tertile 3 | -5.48(-7.73,-3.22) | <0.001 | -0.18(-0.25,-0.11) | <0.001 |
| *P* for trend | <0.001 |  | <0.001 |  |

Adjusted for age, marital status, education levels, smoking, drinking, and vigorous or moderate physical activity.

| Supplementary Table 3. The associations between relative handgrip strength and lipid profiles | | | | | | | | |
| --- | --- | --- | --- | --- | --- | --- | --- | --- |
| Grip strength | TC | | HDLC | | LDLC | | TG | |
|  | β(95%CI) | *P* value | β(95%CI) | *P* value | β(95%CI) | *P* value | β(95%CI) | *P* value |
| **Males** |  |  |  |  |  |  |  |  |
| Tertile 1 | Reference |  | Reference |  |  |  | Reference |  |
| Tertile 2 | 2.11(-1.18,5.39) | 0.209 | 1.62(0.46,2.77) | 0.006 | 0.81(-1.90,3.53) | 0.558 | -4.93(-12.88,3.02) | 0.224 |
| Tertile 3 | 1.27(-2.24,4.78) | 0.479 | 4.01(2.77,5.24) | <0.001 | -0.18(-3.07,2.71) | 0.905 | -18.63(-27.07,-10.19) | <0.001 |
| *P* for trend | 0.476 |  | <0.001 |  | 0.906 |  | <0.001 |  |
| **Females** |  |  |  |  |  |  |  |  |
| Tertile 1 | Reference |  | Reference |  | Reference |  | Reference |  |
| Tertile 2 | 2.11(-1.18,5.39) | 0.209 | 0.89(-0.14,1.91) | 0.089 | 2.64(0.03,5.25) | 0.047 | -2.14(-9.91,5.63) | 0.589 |
| Tertile 3 | 1.27(-2.24,4.78) | 0.479 | 1.84(0.77,2.90) | 0.001 | -1.03(-3.75,1.70) | 0.456 | -20.52(-28.57,-12.48) | <0.001 |
| *P* for trend | 0.228 |  | 0.001 |  | 0.151 |  | <0.001 |  |

Adjusted for age, marital status, education levels, smoking, drinking, and vigorous or moderate physical activity.

TC=total cholesterol, TG=triglyceride, HDLC= high density lipoprotein cholesterol, and LDLC=low density lipoprotein cholesterol

| Supplementary Table 4. Area under the receiver-operating characteristics curve of maximal relative handgrip strength | | | | | |
| --- | --- | --- | --- | --- | --- |
|  | AUC | 95%CI | Cutoff | Sensitivity (%) | Specificity (%) |
| **Males** |  |  |  |  |  |
| Hypertension | 0.57 | 0.54-0.59 | 1.85 | 0.73 | 0.39 |
| Diabetes | 0.59 | 0.55-0.63 | 1.68 | 0.61 | 0.56 |
| Dyslipidemia | 0.54 | 0.52-0.56 | 1.68 | 0.50 | 0.56 |
| **Females** |  |  |  |  |  |
| Hypertension | 0.58 | 0.55-0.60 | 1.13 | 0.62 | 0.51 |
| Diabetes | 0.60 | 0.56-0.63 | 1.31 | 0.86 | 0.29 |
| Dyslipidemia | 0.54 | 0.52-0.57 | 1.27 | 0.74 | 0.33 |
| AUC = Area under the curve | | | | | |

| Supplementary Table 5. The associations between handgrip strength and cardiometabolic disorders (Sensitivity analyses 1) | | | | | | |
| --- | --- | --- | --- | --- | --- | --- |
| Grip strength | Hypertension | | Diabetes | | Dyslipidemia | |
|  | OR(95%CI) | *P* value | OR(95%CI) | *P* value | OR(95%CI) | *P* value |
| **Males** |  |  |  |  |  |  |
| Mean relative handgrip strength |  |  |  |  |  |  |
| Tertile 1 | Reference |  | Reference |  | Reference |  |
| Tertile 2 | 1.06(0.82.-1.37) | 0.64 | 0.69(0.11.-4.23) | 0.684 | 0.89(0.71.-1.11) | 0.283 |
| Tertile 3 | 0.73(0.55.-0.98) | 0.034 | 0.48(0.06.-3.91) | 0.495 | 0.64(0.50.-0.81) | <0.001 |
| *P* for trend | 0.043 |  | 0.488 |  | <0.001 |  |
| Mean absolute handgrip strength* |  |  |  |  |  |  |
| Tertile 1 | Reference |  | Reference |  | Reference |  |
| Tertile 2 | 0.98(0.75.-1.27) | 0.862 | 0.81(0.11.-5.85) | 0.831 | 1.04(0.83-1.32) | 0.716 |
| Tertile 3 | 0.93(0.69.-1.25) | 0.624 | 1.00(0.13.-7.92) | 0.997 | 1.38(1.08-1.77) | 0.010 |
| *P* for trend | 0.626 |  | 0.995 |  | 0.009 |  |
| Maximal absolute handgrip strength* |  |  |  |  |  |  |
| Tertile 1 | Reference |  | Reference |  | Reference |  |
| Tertile 2 | 1.05(0.81-1.38) | 0.694 | 0.84(0.11-6.27) | 0.867 | 1.08(0.85-1.37) | 0.516 |
| Tertile 3 | 0.95(0.71-1.28) | 0.749 | 1.09(0.14-8.81) | 0.935 | 1.35(1.05-1.74) | 0.018 |
| *P* for trend | 0.757 |  | 0.935 |  | 0.016 |  |
| **Females** |  |  |  |  |  |  |
| Mean relative handgrip strength |  |  |  |  |  |  |
| Tertile 1 | Reference |  | Reference |  | Reference |  |
| Tertile 2 | 0.89(0.69.-1.15) | 0.381 | 0.82(0.61.-1.10) | 0.19 | 0.95(0.74.-1.22) | 0.694 |
| Tertile 3 | 0.65(0.49.-0.86) | 0.003 | 0.50(0.36.-0.71) | 0 | 0.69(0.47.-1.00) | 0.048 |
| *P* for trend | 0.003 |  | <0.001 |  | 0.049 |  |
| Mean absolute handgrip strength* |  |  |  |  |  |  |
| Tertile 1 | Reference |  | Reference |  | Reference |  |
| Tertile 2 | 1.23(0.94.-1.60) | 0.133 | 0.91(0.66.-1.25) | 0.549 | 0.82(0.61-1.09) | 0.173 |
| Tertile 3 | 1.20(0.89.-1.61) | 0.232 | 0.99(0.70.-1.39) | 0.941 | 0.98(0.76-1.28) | 0.909 |
| *P* for trend | 0.213 |  | 0.912 |  | 0.902 |  |
| Maximal absolute handgrip strength* |  |  |  |  |  |  |
| Tertile 1 | Reference |  | Reference |  | Reference |  |
| Tertile 2 | 0.98(0.75-1.28) | 0.901 | 0.85(0.62-1.18) | 0.338 | 1.08(0.85-1.37) | 0.516 |
| Tertile 3 | 1.10(0.82-1.47) | 0.522 | 1.02(0.73-1.43) | 0.924 | 1.35(1.05-1.74) | 0.018 |
| *P* for trend | 0.541 |  | 0.972 |  | 0.857 |  |

Adjusted for age, marital status, education levels, smoking, drinking, vigorous or moderate physical activity.

*Body mass index was adjusted for in the model

| Supplementary Table 6. The association between handgrip strength and cardiometabolic disorders (Sensitivity analyses 2#) | | | | | | |
| --- | --- | --- | --- | --- | --- | --- |
| Grip strength | Hypertension | | Diabetes | | Dyslipidemia | |
|  | OR(95%CI) | *P* value | OR(95%CI) | *P* value | OR(95%CI) | *P* value |
| **Males** |  |  |  |  |  |  |
| Quartile1 | Reference |  | Reference |  | Reference |  |
| Quartile2 | 1.08(0.81.-1.44) | 0.612 | 0.76(0.10.-5.51) | 0.782 | 0.83(0.64.-1.07) | 0.147 |
| Quartile3 | 0.99(0.74.-1.34) | 0.956 | 0.44(0.04.-4.57) | 0.495 | 0.71(0.55.-0.93) | 0.012 |
| Quartile4 | 0.61(0.43.-0.86) | 0.004 | 0.51(0.05.-5.28) | 0.575 | 0.57(0.43.-0.75) | <0.001 |
| *P* for trend | 0.008 |  | 0.491 |  | <0.001 |  |
| **Females** |  |  |  |  |  |  |
| Quartile1 | Reference |  | Reference |  | Reference |  |
| Quartile2 | 1.00(0.75.-1.34) | 0.975 | 0.80(0.57.-1.12) | 0.192 | 0.94(0.71.-1.25) | 0.672 |
| Quartile3 | 0.71(0.52.-0.96) | 0.028 | 0.75(0.53.-1.06) | 0.099 | 0.77(0.54.-1.09) | 0.143 |
| Quartile4 | 0.70(0.51.-0.97) | 0.031 | 0.35(0.23.-0.54) | <0.001 | 0.59(0.36.-0.96) | 0.033 |
| *P* for trend | 0.006 |  | <0.001 |  | 0.030 |  |

Adjusted for age, marital status, education levels, smoking, drinking, vigorous or moderate physical activity.

*Body mass index was adjusted for in the model

# Handgrip strength or index were divided into four groups according to the quartiles

| eTable 7. The association between handgrip strength and cardiometabolic disorders (Sensitivity analyses 3#) | | | | | | |
| --- | --- | --- | --- | --- | --- | --- |
| Grip strength | Hypertension | | Diabetes | | Dyslipidemia | |
|  | OR(95%CI) | *P* value | OR(95%CI) | *P* value | OR(95%CI) | *P* value |
| **Males** |  |  |  |  |  |  |
| Tertile 1 | Reference |  | Reference |  | Reference |  |
| Tertile 2 | 1.08(0.81-1.43) | 0.60 | 0.73(0.09-5.96) | 0.767 | 0.97(0.76-1.25) | 0.822 |
| Tertile 3 | 0.69(0.50-0.95) | 0.022 | 0.51(0.05-5.72) | 0.588 | 0.67(0.51-0.88) | 0.004 |
| *P* for trend | 0.028 |  | 0.583 |  | 0.004 |  |
| **Females** |  |  |  |  |  |  |
| Tertile 1 | Reference |  | Reference |  | Reference |  |
| Tertile 2 | 0.85(0.64-1.14) | 0.280 | 0.83(0.59-1.16) | 0.268 | 0.88(0.63-1.22) | 0.442 |
| Tertile 3 | 0.54(0.39-0.75) | <0.001 | 0.50(0.34-0.73) | <0.001 | 0.65(0.41-1.04) | 0.070 |
| *P* for trend | <0.001 |  | 0.001 |  | 0.070 |  |

Adjusted for age, marital status, education levels, smoking, drinking, vigorous or moderate physical activity.

*Body mass index was adjusted for in the model

#1,074 participants with self-reported stroke, chronic lung diseases, depressive symptom (the 10-item Center for Epidemiological Studies–Depression Scale scored 12 points or more), or coronary heart diseases were excluded
